# Consensus transcriptional signature of dengue virus infection reveals coordinated interferon suppression and stress-response activation

**DOI:** 10.1007/s12013-026-02094-0

**Published:** 2026-06-08

**Authors:** Cezar Rangel Pestana, Gerson Jhonatan Rodrigues

**Affiliations:** 1 Latin American Institute of Life and Natural Sciences, Federal University for Latin American Integration, Foz do Iguassu, Brazil; 2 Department of Physiological Sciences, Federal University of Sao Carlos, Sao Carlos, Brazil

**Keywords:** Dengue infection, Gene expression, Drug repurposing, Systems biology

## Abstract

Dengue virus infection remains a major global health threat, with severe dengue disease progressing to life-threatening hemorrhage and shock syndrome. While clinical manifestations are well-documented, the transcriptional landscape associated with dengue disease severity remains incompletely understood. A multi-cohort transcriptomic analysis of public datasets was performed to establish a consensus gene expression signature of dengue virus infection. Functional enrichment and protein–protein interaction networks were used to identify biological processes and highly connected nodes. In silico drug–gene interaction analysis was conducted to prioritize candidate compounds. Clinical validation was performed using an independent cohort to assess gene expression patterns across dengue disease severity groups. Differential expression analysis revealed downregulation of interferon-related genes, including STAT1-associated pathways, alongside upregulation of stress-response genes such as CDKN1A (p21), FOS, and JUNB. Network analysis identified these genes as highly connected nodes, suggesting coordinated interplay between antiviral signaling and endothelial stress responses. Candidate compounds, including Garcinol, Paclitaxel, and Romidepsin, were identified based on curated drug–gene interactions as potential modulators of this transcriptional signature. This study defines a consensus transcriptional signature associated with dengue virus infection and dengue disease severity, highlighting coordinated changes in highly connected genes involved in host response pathways. These findings provide a framework for future experimental validation and the development of host-directed therapeutic strategies.

**Supplementary Information:**

The online version contains supplementary material available at https://doi.org/10.1007/s12013-026-02094-0.

## Introduction

Dengue virus (DENV) is the most prevalent human arbovirus, responsible for significant hemodynamic disturbances in tropical and subtropical regions [1–3]. Transmission primarily occurs via the Aedes aegypti mosquito, leading to infection of macrophages, dendritic cells, and endothelial cells [4]. Clinical manifestations of dengue disease range from a self-limited febrile illness to severe forms characterized by plasma leakage, hemorrhage, and multi-organ failure [5]. The risk of progression to severe dengue disease is multifactorial, influenced by viral serotype, host genetic background, and immune status, particularly during secondary infections with heterologous serotypes [6].

The vascular endothelium serves as a critical semi-permeable barrier regulating fluid homeostasis and paracellular transport [7]. In severe dengue disease, the viral non-structural protein 1 (NS1) directly interacts with the endothelial glycocalyx, triggering endothelial activation and junctional disruption. This interaction leads to a reduction in transendothelial electrical resistance (TEER), resulting in the hallmark plasma leakage observed in severe dengue disease [8]. Due to its pivotal role in these processes, human umbilical vein endothelial cells (HUVECs) are widely used as an in vitro model to investigate molecular mechanisms underlying vascular permeability and host transcriptional responses during dengue virus infection.

Endothelial instability in dengue virus infection is driven by complex interactions between antiviral signaling, inflammatory responses, and cellular stress pathways. STAT1 is a key mediator of type I interferon signaling, and its dysregulation may impair antiviral defense [9]. This mechanism highlights the broader disruption of interferon pathways during dengue virus infection and provides important context for interpreting alterations in STAT1-related signaling.

Similarly, CDKN1A (p21) is widely recognized as a regulator of cell cycle arrest under stress conditions [10], while members of the AP-1 transcription factor family, including FOS and JUNB, are involved in early stress-response and inflammatory signaling pathways [11]. In this context, these genes should not be considered as established drivers of dengue virus pathogenesis, but rather as candidate regulatory nodes that may contribute to endothelial dysfunction and progression of dengue disease, as suggested by systems-level analyses.

This study aims to employ a meta-analytical systems biology approach to identify a consensus molecular signature associated with dengue virus infection and dengue disease severity. By integrating transcriptomic data across multiple datasets, we seek to identify robust gene signatures and regulatory hubs that may improve the understanding of host responses to infection and provide a framework for hypothesis generation in host-directed therapeutic strategies targeting vascular dysfunction.

## Methods

### Data Acquisition and Differential Expression Analysis

Transcriptomic datasets (GSE9378, GSE34628, and GSE139603) were retrieved from the Gene Expression Omnibus (GEO) database [4, 12, 13]. These datasets were selected to capture complementary aspects of the host response to dengue virus infection: GSE139603 represents endothelial responses to NS1 stimulation, GSE34628 represents direct dengue virus infection in endothelial cells, and GSE9378 captures broader host responses across multiple cell types. Raw expression data were subjected to background correction and quantile normalization using the limma package in R. Differential expression analysis was performed using linear models with empirical Bayes moderation, applying a threshold of log₂ fold change > 0.5 and adjusted p-value < 0.05 using the Benjamini–Hochberg method.

To minimize batch effects and increase robustness, a consensus-based integration strategy was employed, retaining only genes that were consistently differentially expressed across all datasets with concordant directionality. This approach prioritizes reproducible signals across heterogeneous experimental conditions rather than relying on direct data merging. Visualization of differential expression results was performed using VolcanoseR, and hierarchical clustering with heatmap representation was conducted using Morpheus (Broad Institute).

### Consensus Signature and Intersection Analysis

To define a robust molecular signature associated with dengue virus infection and dengue disease severity, an integrative cross-dataset analysis was performed by intersecting differentially expressed genes identified in all three cohorts. Venn diagram analysis was used to identify overlapping upregulated and downregulated genes. Only genes with consistent directionality across datasets were retained to construct the consensus signature, reducing the influence of dataset-specific variability [14].

### Network Topology and Functional Enrichment

Consensus differentially expressed genes were subjected to functional enrichment analysis using Enrichr with MSigDB gene sets, applying False Discovery Rate (FDR) correction [15, 16]. Combined scores were used as an integrated metric reflecting both statistical significance and enrichment magnitude. Gene Ontology (GO) terms were additionally evaluated. Protein–protein interaction (PPI) networks were constructed using STRING-db (version 11.5) [17], considering only high-confidence interactions (interaction score > 0.700). Network topology analysis was performed to identify hub genes based on degree centrality, with particular focus on STAT1, CDKN1A, FOS, and JUNB. Drug–gene interactions were retrieved from the Drug–Gene Interaction Database (DGIdb, version 4.0). Candidate compounds were identified by querying DGIdb based on curated and predicted drug–gene interactions [18]. Interactions were prioritized using aggregated evidence scores reflecting interaction strength, number of gene targets, and relevance to the identified transcriptional signature.

### Clinical Validation and Statistical Analysis

**Fig. 1:**
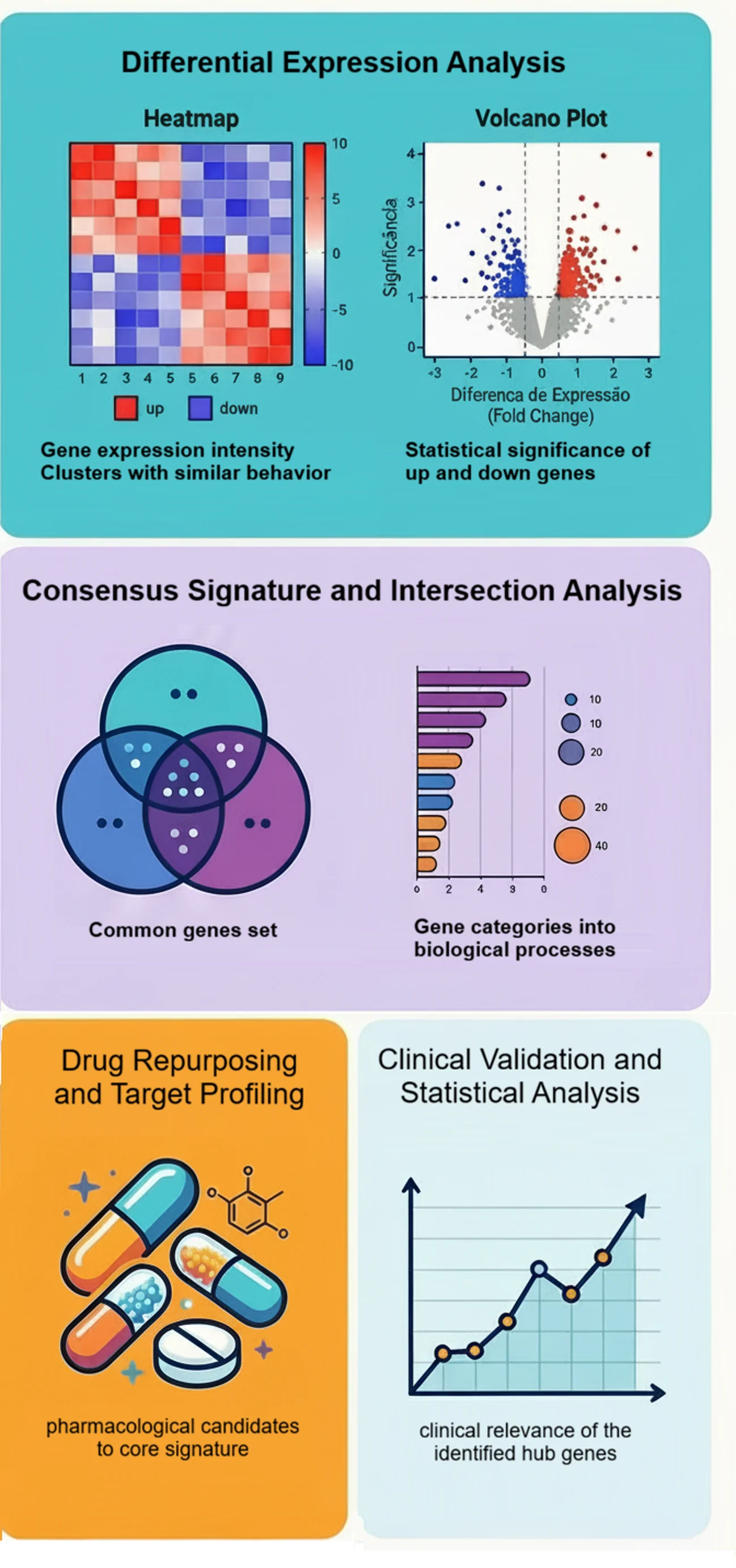
Study Workflow. Schematic representation of the integrated bioinformatic workflow. The study is divided into four main stages: Data acquisition and preprocessing of transcriptomic datasets from the GEO database; Differential Expression Analysis to identify significant up-regulated and down-regulated genes; Intersection and Network Analysis to discover core genes and biological processes via GO enrichment; and Drug repurposing and clinical validation of identified hub genes

The clinical relevance of the identified hub genes was evaluated using an independent transcriptomic cohort (GSE17924), comprising patients stratified into dengue disease severity groups: Dengue Fever (DF), Dengue Hemorrhagic Fever (DHF), and Dengue Shock Syndrome (DSS). Gene expression differences across clinical groups were assessed using one-way ANOVA followed by Tukey’s post-hoc test. Statistical significance was defined as p-value < 0.05. Receiver operating characteristic (ROC) curve analysis was performed to evaluate the discriminatory performance of individual hub genes between severity groups (DF vs. DSS and DHF vs. DSS), with DSS defined as the positive class. The area under the curve (AUC), sensitivity, and specificity were calculated using optimal thresholds determined by Youden’s index. Confidence intervals (95%) for AUC were estimated using bootstrap resampling (1,000 iterations). Given the limited sample size, internal validation was performed using leave-one-out cross-validation (LOOCV), in which each sample was iteratively excluded and classification performance recalculated. This approach was used to assess robustness and mitigate potential overestimation of predictive performance. The overall analytical workflow is summarized in Fig. 1.

## Results

### Suppression of Interferon Signaling and Activation of Cellular Stress Pathways

**Fig. 2:**
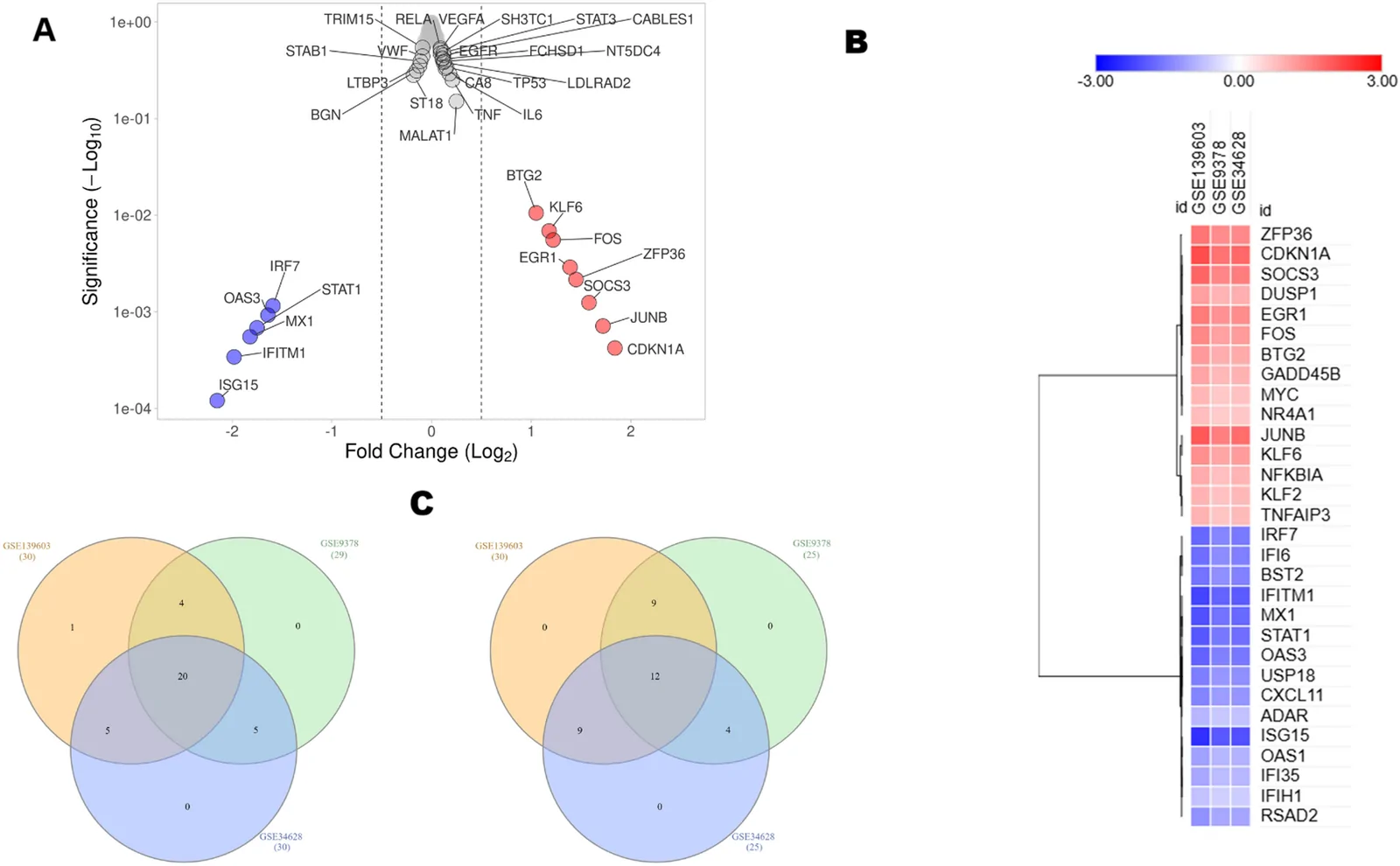
Differential expression and intersection analysis. Identification and integration of differentially expressed genes across datasets. (A) Volcano plots illustrating the results of differential expression analysis, where each point represents a gene plotted according to log₂ fold change and adjusted p-value. Red dots indicate significantly upregulated genes and blue dots indicate significantly downregulated genes (|log₂FC| > 0.5; adjusted p-value < 0.05). (B) Heatmap of consensus DEGs showing relative expression patterns across datasets based on hierarchical clustering (red = upregulation; blue = downregulation). (C) Venn diagrams representing the intersection of DEGs across datasets, identifying a core set of commonly upregulated (*n* = 12) and downregulated (*n* = 20) genes used for subsequent analyses

To systematically characterize the transcriptional landscape associated with dengue infection, we performed an integrative multi-step analysis combining differential gene expression, cross-dataset consensus identification, network topology and functional enrichment approaches. Differential expression analysis was independently performed for each dataset using a threshold of log₂ fold change > 0.5 and adjusted p-value < 0.05. The distribution of differentially expressed genes (DEGs), their intersection across datasets, and associated expression patterns are illustrated in Fig. 2.

Volcano plots (Fig. 2A) highlight significantly upregulated and downregulated genes based on fold change and statistical significance, revealing a consistent pattern across datasets characterized by downregulation of antiviral genes and upregulation of stress- and inflammation-related genes. These patterns are further supported by hierarchical clustering heatmaps (Fig. 2B), which show coherent expression profiles of consensus DEGs across datasets.

Intersection analysis identified a consensus transcriptional signature comprising genes consistently altered across all datasets despite differences in experimental systems. Only genes displaying concordant directionality were retained, ensuring robustness of the identified signature (Fig. 2C). Notably, key interferon-stimulated genes (ISGs), including ISG15, OAS3, and MX1, were consistently downregulated, supporting coordinated suppression of antiviral responses during dengue virus infection. In contrast, genes associated with cellular stress responses and inflammation were predominantly upregulated, including CDKN1A, FOS, and JUNB.

### STAT1 and CDKN1A as Central Regulatory Hubs of Dengue Virus Infection

**Fig. 3:**
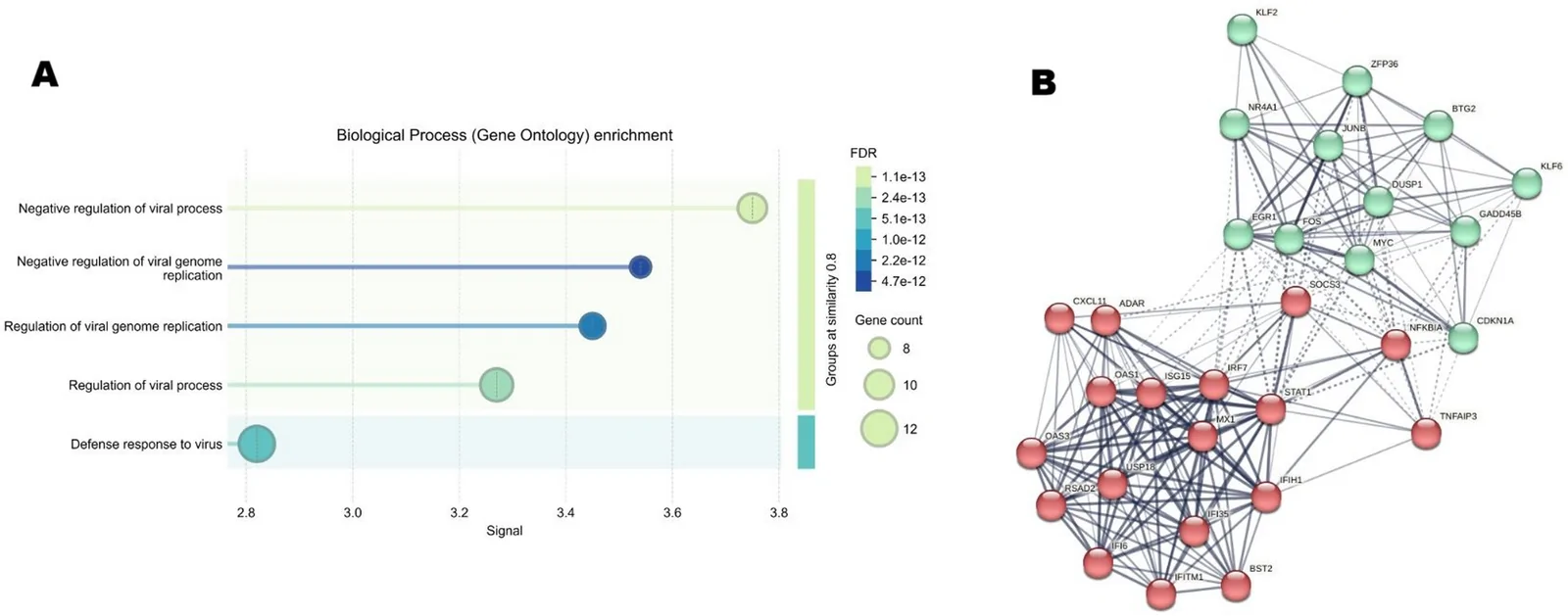
Functional enrichment and protein interaction analysis. (A) Functional enrichment analysis of consensus differentially expressed genes performed using MSigDB Hallmark gene sets through the Enrichr platform. Enriched pathways are ranked according to the Combined Score, calculated as the product of the log-transformed p-value and the z-score deviation from the expected rank. Interferon-related pathways (e.g., Interferon Alpha Response, Interferon Gamma Response) are prominently enriched among downregulated genes, whereas inflammatory pathways (e.g., TNF-α signaling via NF-κB) are enriched among upregulated genes. Bubble size represents gene count, and color indicates False Discovery Rate (FDR). (B) Protein–protein interaction (PPI) network generated using the STRING database (v11.5), displaying high-confidence interactions (interaction score > 0.700) among consensus DEGs and highlighting highly connected hub genes

Functional enrichment analysis demonstrated that downregulated genes were strongly enriched in Interferon Alpha Response (Combined Score: 76,742) and Interferon Gamma Response (Combined Score: 44,119) pathways. In contrast, upregulated genes exhibited marked enrichment in TNF-α signaling via NF-κB (Combined Score: 1.3 × 10⁷) and hypoxia-related pathways (Fig. 3A). Protein–protein interaction (PPI) network analysis (Fig. 3B) revealed distinct modules, with the downregulated cluster centered on antiviral regulators such as STAT1, IRF7, and MX1, and the upregulated cluster including CDKN1A, FOS, and JUNB, associated with cell cycle regulation and stress-response pathways. Network topology analysis identified STAT1 and CDKN1A as highly connected nodes based on degree centrality, supporting their role as central components of the transcriptional response to dengue virus infection.

### Mapping Candidate Modulators of the Core Signature

Based on the identified hub genes, in silico drug–gene interaction analysis was performed to identify candidate modulators of the transcriptional signature. Paclitaxel emerged as a multi-target compound associated with both FOS and CDKN1A. Garcinol was linked to STAT1-associated regulatory networks, while Romidepsin and 4-Phenylbutyric acid (4-PBA) were identified based on interactions involving CDKN1A-related mechanisms. These interactions are derived from curated databases and should be interpreted as hypothesis-generating rather than indicative of direct therapeutic efficacy. The resulting drug–gene interaction landscape is summarized in Table 1.

**Table 1:** Candidate compounds identified through drug–gene interaction analysis

Drug candidate	Gene target	Association score	Category
Garcinol	STAT1	2.175158	Immune response
Ipriflavone	STAT1	0.725053	Immune response
4-phenylbutyric acid	CDKN1A	0.168399	Cell survival
Romidepsin	CDKN1A	0.120285	Cell survival
Paclitaxel	FOS	0.036506	Cell cycle
Paclitaxel	CDKN1A	0.030618	Cell cycle

Interactions were retrieved from the DGIdb based on curated and predicted evidence. Compounds were prioritized according to aggregated interaction scores reflecting the strength and number of gene–drug associations within the consensus transcriptional signature

### Hub Gene Expression Stratifies Dengue Disease Severity

**Fig. 4:**
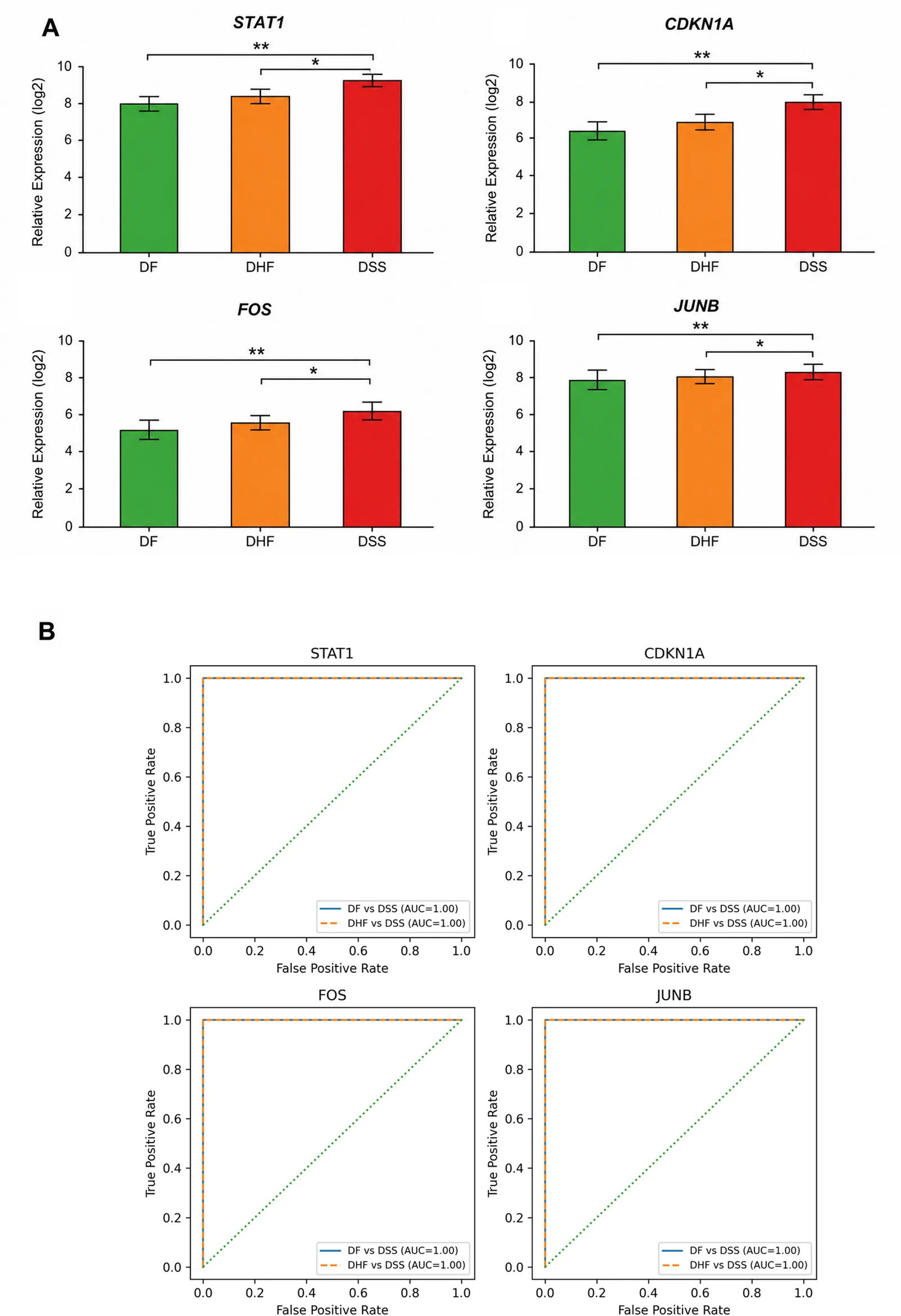
Clinical validation of hub gene expression across dengue severity groups. (A) Expression levels of STAT1, CDKN1A, FOS and JUNB in DF, DHF and DSS patients. Data are presented as mean ± standard error of the mean (SEM). Statistical comparisons were performed using one-way ANOVA followed by Tukey’s post-hoc test for multiple comparisons. Asterisks indicate statistically significant differences between groups (*P < 0.05, **P < 0.01). (B) ROC curve analysis for STAT1, CDKN1A, FOS and JUNB performed in discriminating dengue severity groups. Solid lines represent DF vs. DSS comparisons, and dashed lines represent DHF vs. DSS comparisons. The diagonal dotted line indicates random classification. Analyses were performed using *n* = 5 samples per group

Validation using the GSE17924 cohort demonstrated that the identified core signature is associated with dengue disease severity (Fig. 4A). Gene expression levels were compared across Dengue Fever (DF), Dengue Hemorrhagic Fever (DHF), and Dengue Shock Syndrome (DSS) groups using one-way ANOVA followed by Tukey’s post-hoc test. STAT1 expression was significantly increased in DSS compared to DF, in contrast to its downregulation observed in endothelial in vitro models. CDKN1A and FOS showed progressive upregulation from DF to DHF and DSS, indicating a gradual increase in stress-related gene expression with increasing dengue disease severity. JUNB expression was significantly elevated in DSS compared to both DF and DHF. Individual expression values for all samples are provided in Supplementary Table S1.

Receiver operating characteristic (ROC) curve analysis demonstrated high discriminatory performance for all hub genes in distinguishing both DF vs. DSS and DHF vs. DSS groups (Fig. 4B). All genes achieved an area under the curve (AUC) of 1.00, with corresponding sensitivity and specificity values of 1.00. These results reflect clear separation between clinical groups in the dataset. However, given the limited sample size (*n* = 5 per group), confidence intervals were broad, and these findings should be interpreted with caution. Internal validation using leave-one-out cross-validation (LOOCV) yielded consistent results, although the potential for overestimation of predictive performance cannot be excluded. Detailed ROC performance metrics are provided in Supplementary Table S2.

To further assess consistency across datasets, dataset-specific expression patterns of key hub genes were examined. STAT1 was consistently downregulated across all discovery datasets, including endothelial NS1 stimulation and viral infection models. In contrast, the independent clinical cohort (GSE17924) showed increased STAT1 expression in patients with severe dengue disease (DSS) compared to DF and DHF groups. This divergence likely reflects differences in biological context, including cell type–specific responses and the distinction between localized endothelial models and systemic immune responses captured in whole-blood transcriptomic data.

## Discussion

This study presents an integrated transcriptomic analysis of publicly available datasets to identify a consensus molecular signature associated with dengue virus infection severity. By intersecting differentially expressed genes across distinct experimental systems, we identified a robust gene set characterized by downregulation of interferon-related pathways and upregulation of stress and inflammatory responses. Network-based analysis further highlighted STAT1 and CDKN1A as highly connected nodes within this signature, suggesting their potential relevance in the host response to dengue virus infection.

Our findings indicate that dengue virus infection is associated with a coordinated transcriptional reprogramming involving activation of TNF-α/NF-κB signaling alongside modulation of interferon pathways, consistent with previous reports describing the interplay between antiviral defense and inflammatory activation [9, 19]. While several studies have reported induction of interferon-stimulated genes (ISGs) during dengue virus infection, the observed downregulation of ISGs in our analysis likely reflects experimental differences related to the use of NS1 stimulation, which does not fully recapitulate viral replication, as well as differences in sampling timepoints and cellular models [20]. Additionally, interferon signaling is highly dynamic, with early antiviral responses potentially followed by compensatory or dysregulated activation at later stages of infection. These observations highlight the complexity and temporal heterogeneity of interferon responses during dengue virus infection. STAT2 also plays a critical role in type I interferon signaling and is a well-established target of dengue virus immune evasion. The viral NS5 protein has been shown to mediate degradation of STAT2, thereby impairing interferon signaling and facilitating viral replication [21]. The role of STAT2 should also be considered in future analyses to provide a more comprehensive view of interferon pathway modulation.

The identification of CDKN1A as a central upregulated node suggests a potential mechanistic link between cellular stress response and endothelial dysfunction. Its enrichment in hypoxia-related pathways raises the possibility that endothelial cells may undergo stress-associated phenotypic changes, potentially including features consistent with premature senescence [10]. Such alterations have been associated with impaired barrier function and increased vascular permeability in previous studies [8]. In parallel, the upregulation of transcription factors such as FOS and JUNB suggests activation of early-response regulatory programs involved in stress adaptation and inflammatory signaling [11]. While these genes have been implicated in endothelial responses to stress, their precise role in dengue virus infection pathogenesis remains to be fully elucidated.

The identification of hub genes based on network topology does not establish causal regulatory roles. Instead, these genes should be interpreted as highly connected nodes within the transcriptional network, whose functional relevance requires further experimental validation. Validation using the independent GSE17924 cohort supported the association between hub gene expression and clinical severity. The differential expression patterns observed across Dengue Fever (DF), Dengue Hemorrhagic Fever (DHF), and Dengue Shock Syndrome (DSS) groups suggest that CDKN1A and FOS are involved in early stress-related responses, whereas STAT1 and JUNB are more prominently associated with severe disease stages. These findings likely reflect systemic immune activation captured in whole-blood samples and highlight the importance of considering tissue- and context-specific differences when interpreting transcriptomic data.

In addition to differential expression patterns, ROC curve analysis demonstrated that all evaluated hub genes (STAT1, CDKN1A, FOS, and JUNB) exhibited maximal discriminatory performance (AUC = 1.00) in distinguishing both DF vs. DSS and DHF vs. DSS groups. While these results reflect the clear separation observed between clinical groups in the analyzed dataset, they should be interpreted with caution. The limited sample size (*n* = 5 per group) and low intra-group variability likely contribute to overestimation of predictive performance, a well-recognized limitation in small-cohort transcriptomic studies. Confidence intervals were broad despite perfect classification, reinforcing the need for cautious interpretation. Internal validation using leave-one-out cross-validation yielded consistent results; however, independent validation in larger cohorts will be essential to confirm the robustness and generalizability of these candidate biomarkers.

A notable finding of this study is the divergence in STAT1 expression between experimental models and the clinical cohort. While STAT1 was consistently downregulated across all discovery datasets, including endothelial NS1 stimulation and viral infection models, it was significantly upregulated in patients with severe disease in the independent clinical cohort. This discrepancy likely reflects differences in biological context. Endothelial models primarily capture localized cellular responses to viral components, where suppression of interferon signaling may facilitate viral immune evasion. In contrast, whole-blood transcriptomic profiles represent systemic immune responses, where increased STAT1 expression may reflect compensatory or dysregulated interferon activation associated with severe disease. These findings highlight the context-dependent nature of interferon signaling during dengue virus infection and underscore the importance of integrating data from complementary biological systems when interpreting transcriptomic signatures.

Based on these central hub genes, we explored potential pharmacological modulators using a drug–gene interaction framework. Compounds such as Paclitaxel, Garcinol, Romidepsin, and 4-Phenylbutyric acid were identified as candidates with reported interactions involving key components of the transcriptional signature. Previous studies have shown that Paclitaxel can influence endothelial barrier stability, while Romidepsin modulates inflammatory responses, supporting their potential relevance in this context [22–24]. In the context of our analysis, their association with CDKN1A-centered networks supports their potential biological relevance in endothelial and inflammatory regulation [25]. However, these associations are based on curated interaction data and do not account for pharmacokinetic properties, dose-response relationships, or disease-specific effects. Therefore, these findings should be interpreted as a framework for hypothesis generation, requiring further experimental and clinical validation.

The integration of datasets representing distinct biological systems and experimental conditions provides a broad view of host responses to dengue virus infection but also introduces important interpretative considerations. While this approach enables the identification of conserved transcriptional patterns across studies, it may not fully capture context-specific regulatory mechanisms. Therefore, the resulting gene signature should be interpreted as a cross-context representation of host response rather than a strictly uniform disease-specific profile.

In addition, this study is based entirely on in silico analyses, and several methodological constraints should be considered. Variability arising from differences in cell types, experimental designs, and analytical platforms may influence the observed transcriptional patterns, despite normalization and integration strategies. Furthermore, the identification of hub genes is based on network topology and does not establish causal relationships. In particular, the near-perfect discriminatory performance observed in ROC analyses should be interpreted in the context of the small sample size and requires validation in larger, independent cohorts before any clinical translation can be considered. While these findings provide a framework for understanding host responses in dengue virus infection, further experimental and clinical studies are required to validate these observations.

## Conclusion

This study provides an integrative transcriptomic analysis identifying a consensus molecular signature associated with dengue virus infection severity. By combining multiple experimental datasets with clinical validation, we highlight conserved gene networks characterized by suppression of interferon-related pathways and activation of stress-response and inflammatory mechanisms. STAT1, CDKN1A, FOS, and JUNB, were consistently identified as central components of this signature. Their differential expression across clinical severity groups supports their potential relevance in disease progression, although context-dependent differences underscore the complexity of host responses to dengue virus infection. Finally, the identification of candidate compounds provides a preliminary framework for hypothesis generation in host-directed therapeutic strategies.

## Supplementary Material

Below is the link to the electronic supplementary material.

Supplementary Material 1

Supplementary Material 2

## Data Availability

The data that support the findings of this study are available in the NCBI Gene Expression Omnibus (GEO) at https://www.ncbi.nlm.nih.gov/geo/, accession reference numbers GSE139603, GSE9378, GSE34628, and GSE17924.
