# Supplementary material for: Consensus transcriptional signature of dengue virus infection reveals coordinated interferon suppression and stress-response activation
Source: Cell Biochem Biophys. 2026 Jun 8;84(3):3899–908. doi: 10.1007/s12013-026-02094-0 (PMC13585856; doi:10.1007/s12013-026-02094-0)
Supplement: Supplementary file 1 [file 12013_2026_2094_MOESM1_ESM.docx]

## Table S1. Individual gene expression values across clinical severity groups.

| Sample ID | Clinical Group | STAT1 | CDKN1A | FOS | JUNB |
| --- | --- | --- | --- | --- | --- |
| GSM447958 | DSS | 9.42 | 7.95 | 6.15 | 8.21 |
| GSM447959 | DSS | 9.38 | 8.10 | 6.22 | 8.18 |
| GSM447960 | DSS | 9.50 | 7.88 | 6.05 | 8.25 |
| GSM447961 | DSS | 9.25 | 8.02 | 6.11 | 8.10 |
| GSM447962 | DSS | 9.44 | 7.91 | 6.18 | 8.23 |
| GSM447977 | DF | 8.05 | 6.35 | 5.10 | 7.82 |
| GSM447978 | DF | 8.12 | 6.42 | 5.18 | 7.88 |
| GSM447979 | DF | 7.98 | 6.28 | 5.05 | 7.75 |
| GSM447980 | DF | 8.18 | 6.50 | 5.22 | 7.91 |
| GSM447981 | DF | 8.02 | 6.31 | 5.12 | 7.84 |
| GSM447993 | DHF | 8.35 | 6.85 | 5.52 | 7.95 |
| GSM447994 | DHF | 8.42 | 6.92 | 5.58 | 7.98 |
| GSM447995 | DHF | 8.29 | 6.78 | 5.45 | 7.91 |
| GSM447996 | DHF | 8.51 | 7.05 | 5.65 | 8.02 |
| GSM447997 | DHF | 8.38 | 6.88 | 5.54 | 7.96 |
