# Supplementary material for: Consensus transcriptional signature of dengue virus infection reveals coordinated interferon suppression and stress-response activation
Source: Cell Biochem Biophys. 2026 Jun 8;84(3):3899–908. doi: 10.1007/s12013-026-02094-0 (PMC13585856; doi:10.1007/s12013-026-02094-0)
Supplement: Supplementary file 2 [file 12013_2026_2094_MOESM2_ESM.docx]

## Table S2. Receiver operating characteristic (ROC) analysis of hub genes for discrimination between clinical severity groups.

| Gene | Comparison | AUC | 95% CI | Sensitivity | Specificity | Optimal Cut-off | n (per group) |
| --- | --- | --- | --- | --- | --- | --- | --- |
| STAT1 | DF vs DSS | 1.00 | 0.57–1.00 | 1.00 | 1.00 | 8.90 | 5 vs 5 |
| STAT1 | DHF vs DSS | 1.00 | 0.57–1.00 | 1.00 | 1.00 | 9.05 | 5 vs 5 |
| CDKN1A | DF vs DSS | 1.00 | 0.57–1.00 | 1.00 | 1.00 | 7.40 | 5 vs 5 |
| CDKN1A | DHF vs DSS | 1.00 | 0.57–1.00 | 1.00 | 1.00 | 7.65 | 5 vs 5 |
| FOS | DF vs DSS | 1.00 | 0.57–1.00 | 1.00 | 1.00 | 5.60 | 5 vs 5 |
| FOS | DHF vs DSS | 1.00 | 0.57–1.00 | 1.00 | 1.00 | 5.85 | 5 vs 5 |
| JUNB | DF vs DSS | 1.00 | 0.57–1.00 | 1.00 | 1.00 | 8.05 | 5 vs 5 |
| JUNB | DHF vs DSS | 1.00 | 0.57–1.00 | 1.00 | 1.00 | 8.12 | 5 vs 5 |
